# The needs and preferences of pregnant smokers regarding tailored Internet-based smoking cessation interventions: a qualitative interview study

**DOI:** 10.1186/1471-2458-14-1070

**Published:** 2014-10-14

**Authors:** Aleksandra Herbec, Emma Beard, Jamie Brown, Benjamin Gardner, Ildiko Tombor, Robert West

**Affiliations:** Cancer Research UK Health Behaviour Research Centre, Department of Epidemiology and Public Health, University College London, Gower Street, London, WC1E 6BT UK; Clinical, Educational and Health Psychology, University College London, 1-19 Torrington Place, London, WC1E 7HB UK; National Centre for Smoking Cessation and Training, London, UK

**Keywords:** Smoking cessation, Pregnancy, Internet, Online, Self-help, Tailoring, Targeting, Qualitative study

## Abstract

**Background:**

Internet-based Smoking Cessation Interventions (ISCIs) may help pregnant smokers who are unable, or unwilling, to access face-to-face stop smoking support. Targeting ISCIs to specific groups of smokers could increase their uptake and effectiveness. The current study explored the needs and preferences of pregnant women seeking online stop smoking support with an aim to identify features and components of ISCIs that might be most attractive to this population.

**Methods:**

We conducted qualitative interviews with thirteen pregnant women who completed the intervention arm of a pilot randomized controlled trial of a novel ISCI for pregnant smokers (‘MumsQuit’). The interviews explored women’s views towards MumsQuit and online support with quitting smoking in general, as well as their suggestions for how ISCIs could be best targeted to pregnancy. Interview transcripts were analyzed using Framework Analysis.

**Results:**

Participants expressed preferences for an accessible, highly engaging and targeted to pregnancy smoking cessation website, tailored to individuals’ circumstances as well as use of cessation medication, offering comprehensive and novel information on smoking and quitting smoking in pregnancy, ongoing support with cravings management, as well as additional support following relapse to smoking. Participants also viewed as important targeting of the feedback and progress reports to baby’s health and development, offering personal support from experts, and providing a discussion forum allowing for communication with other pregnant women wanting to quit .

**Conclusions:**

The present study has identified a number of potential building blocks for ISCIs targeted to quitting smoking in pregnancy. Pregnant smokers willing to try using ISCI may particularly value an engaging intervention offering a high degree of targeting of comprehensive information to them as a group and tailoring support and advice to their individual needs, as well as one providing post-relapse support, peer-to-peer communication and personal support from experts.

**Electronic supplementary material:**

The online version of this article (doi:10.1186/1471-2458-14-1070) contains supplementary material, which is available to authorized users.

## Background

Smoking during pregnancy has adverse outcomes for mothers and infants, including higher risk of miscarriage [1], and birth defects [2]. Prevalence of maternal smoking during pregnancy is estimated at between 10% and 25% in the US [3] and several European countries [4]. In the UK around 12% of pregnant women smoke throughout pregnancy [5]. Most pregnant smokers want to quit [6], but almost half of those who smoke immediately before or during pregnancy do not manage to quit before giving birth [5].

Behavioral support is the most effective smoking cessation intervention for pregnant smokers [7]. In the UK, it is offered through National Health Services (NHS) Stop Smoking Services, and in England 47% of pregnant attendees who set a quit date with the services are biochemically-verified as abstinent at 4-weeks follow-up [8]. Yet, only a small minority of pregnant smokers access them [9]. Barriers reported by pregnant smokers, including time constraints and fear of stigmatization or failure, may be among the key reasons for why pregnant women do not engage with the established services [10, 11].

An alternative cessation support found acceptable to pregnant smokers are self-help interventions, including printed materials [6, 12] and text-messaging [13, 14]. Yet, the vast majority of UK quit attempts remain completely unaided [15].

There is therefore a need to develop new and complementary methods of delivering effective evidence-based cessation support, accommodating the needs and preferences of pregnant smokers. Technology-based or digital interventions are becoming a focus of much recent research, and although not without challenges, they are recognized as a potentially important aspect in future substance use treatment [16]. Moreover, given that 70% of UK smokers have internet access [17], internet-based smoking cessation interventions (ISCIs) - primarily automated, web-based programs that assist users through cessation, may offer a feasible and potentially highly cost-effective option for treatment of smoking in pregnancy, yet have received little attention.

ISCIs may be particularly suitable for pregnant smokers as they can offer anonymous, convenient and immediately available assistance, particularly to women who are not willing or not able to engage with health professionals face-to-face or over the telephone. They also offer a possibility of extending the reach and support offered through traditional treatment [18]. Additionally, given technological advances, ISCIs can increasingly deliver more comprehensive, tailored and interactive support and advice than other forms of self-help interventions. Importantly, interactive and tailored ISCIs can increase relevance [19] and quit rates [20, 21], and tend to be more effective than booklets or e-mail-based interventions [22].

Designing optimally effective interventions requires consideration of possible intervention functions (e.g. coercion, education), delivery mode, and component Behavior Change Techniques (BCTs; e.g. social support, self-monitoring) [23]. Targeting interventions to shared characteristics of user groups could further increase relevance and reach [24, 25], and has been previously used in targeting to adolescents [26] and ethnicity [27]. ISCIs aimed at pregnant smokers should also include features that users will find appealing, acceptable and potentially helpful, which has not yet been researched.

This study sought to explore the needs and preferences of pregnant smokers seeking online stop smoking help to inform future research on the features of ISCIs attractive to this population, which were extracted from responses of women taking part in a pilot trial of a new internet-based cessation intervention, MumsQuit [28]. The study is part of a wider program of research on smoking and quitting in pregnancy, including an ongoing mixed-methods development and evaluation of a package of digital smoking cessation interventions for pregnant women, as informed by the Medical Research Council guidelines [29]. The present article offers potential building blocks for the development of smoking cessation websites that could help pregnant women quit smoking.

## Methods

### Design

Semi-structured qualitative interviews were nested in an ongoing UK-based two-arm pilot randomized controlled trial of a minimally targeted to pregnancy ISCI (‘MumsQuit’). The study was approved by the UCL Research Ethics Committee (3556/002).

### Participants

Views towards ISCIs were elicited from participants who were randomized to the intervention arm of the MumsQuit trial. Trial participants were recruited from among women already seeking information or support with quitting online through advertisements on smoking cessation discussion forums and websites for UK pregnant women, including the NHS Smokefree site devoted to quitting smoking in pregnancy (an updated version of the website is available at http://www.nhs.uk/smokefree/why-quit/smoking-in-pregnancy, accessed on 15 October 2014). Inclusion criteria for the trial were: being pregnant, aged 18 and over, UK-based, interested in making a serious quit attempt, internet access, willingness to be contacted for a telephone follow-up, and consenting to participate online.

All participants randomized to the intervention arm by 1^st^ September 2012 and contactable within five telephone calls between 9–12 weeks post-baseline (n = 33; 50% of the sample enrolled in that period) were invited to participate in the telephone interview. The invitations explained that the interviews would focus on women’s views on online cessation help with an aim to inform future development of more attractive and helpful websites. The right to withdraw from the interview was emphasized, and participants decided about the date and timing for the interview.

### Interviews

Each participants took part in a one-off 35 minute telephone interview conducted by AH, who was blind to the trial outcome data throughout the analysis of the interview data. Interviews followed a flexible semi-structured schedule of open questions exploring 4 broad topics anchored to the context of MumsQuit intervention: reasons for joining the trial, experiences and views on MumsQuit, recommendations for future ISCIs for pregnant women, and views towards ISCIs more generally. Participants were encouraged to discuss topics important to them, and were not asked to comment on a pre-determined list of ISCI features. Participant responses guided interview progression. Impromptu probes (e.g. ‘could you tell me more about this?’, ‘could you explain why this would be important to you?’) were used to invite elaboration. Interview recordings were transcribed by a third party and the transcripts were verified by AH.

### MumsQuit intervention

‘MumsQuit’ intervention constituted an anchor for a broader discussion about attractive features of ISCIs. MumsQuit is an adaptation of ‘StopAdvisor’, a theory- and evidence-based ISCI delivering tailored advice through a structured, 4-week automated program that emulates behavioral support from an expert advisor (for details see [30, 31]). Both interventions are informed by PRIME theory of motivation [32], which addresses key constructs in smoking cessation, including motivation, self-regulatory capacity and skills, and intervention engagement. The theory also outlines the importance of fostering a non-smoker identity, which is a core BCT in smoking cessation [33], because from identity follows identity-congruent motives, self-regulation, and behavioral stability. The treatment strategy used in MumsQuit and StopAdvisor mirrors that offered throughout UK smoking cessation services. MumsQuit differs from StopAdvisor by means of ‘minimal targeting’ to pregnancy, which involved modification of advice on cessation medications (e.g. exclusion of Varenicline and Bupriopion as options, and qualifying advice about nicotine replacement therapy (NRT)), provision of maternal smoking risk information, minimally relating quit progress feedback to baby’s health, and inclusion of images appealing to mother and pregnant ex-smoker identities.

### Data analysis

Participants’ needs and preferences were extrapolated from interview transcripts using a variant of Framework Analysis (FA) [34, 35]. FA is a systematic and transparent method suitable to intervention evaluation using pre-defined samples and themes [36], while also permitting emergence of unforeseen themes [34]. FA involved four main stages: familiarization, identification of recurrent themes and subthemes, development and refinement of a thematic framework through systematic indexing of transcripts, and development of descriptive accounts. FA was applied to all interview data, without placing constraints on the context within which the observations emerged. Given that the present study was exploratory in nature, all issues raised by any of the participants were treated as potentially important to inform future exploration using quantitative methodology. A realist epistemological perspective was adopted [37].

FA was systematically applied to complete interview transcripts. This paper reports only results relating to participants’ ISCI needs and preferences and relevant background information, and not on peripheral themes, such as those relating to women’s pre-pregnancy experiences of quitting. FA was conducted manually by AH using Excel spreadsheets to chart accounts matrixes, with interpretation verified via independent theme generation by EB. Emergent themes and disputes were discussed and resolved with other co-authors. External validation was conducted through respondent validation [34]: participants were posted summaries of findings and a pre-paid envelope, and were encouraged to reply indicating whether our interpretations reflected their experiences. Responses were received from 3 interviewees (23%) stating that they agreed with our interpretations. Internal validity was ensured via constant comparison [37] and deviant case analysis [38].

### Reflexivity

BG is an experienced qualitative researcher, while AH, EB & IT are mixed-methods researchers. Except for IT, the researchers had minimal experience with working with pregnant smokers prior to initiating the research program on digital interventions for pregnant women, of which MumsQuit is a part. However, EB, JB and RW have published extensively on tobacco control, smoking cessation, and health behavior change. Additionally, AH, JB, IT and RW conduct research on developing and evaluating tailored, theory-informed digital interventions for smoking cessation based on taxonomies of behavior change. The authors believe that well-designed and appropriately tailored and targeted digital intervention have potential to support smokers to quit, both as stand-alone interventions, and as aids to traditional treatments. The background of the authors and the context of the wider research program influenced the study design and the analysis by focusing it on gaining insights on how to further develop more tailored interventions for pregnant smokers.

## Results

Thirteen participants (39.3% of invitees) consented and were interviewed. The most common reasons (65%) provided by women for non-participation were lack of time or no interest in the interview study. The interviewed participants were on average 31 years old (range 20–41), 12 (92%) were White/British, 12 (92%) were married, 5 (39%) were multigravidas and 8 (61%) primigravida. Nine women (69%) had tried to quit smoking before. At the time of the interview 7 (54%) reported having quit smoking. On average, interviewees logged in 5.7 times (range 1–18 times), spent 36.6 minutes browsing the website (range 2–204 minutes), and viewed 112 pages (range 8–340 pages). This engagement was greater than among non-interviewees. It is not possible to determine whether the reasons provided for declining to participate in this study, such as time constraints, may also have influenced the levels of website engagement, or whether women’s lack of engagement meant they were less interested in participating in the interviews, but simply cited other reasons.

Two levels of analysis are presented: first, a detailed and descriptive analysis of participants’ specific preferences for ISCI components and features; and second, a synthesis of the findings suggesting what are the core needs underlying participants’ accounts, as identified from the descriptive themes. The descriptive analysis generated six major themes, comprising 17 subthemes, which are described in detail below. These revealed participants’ preferences for ISCIs to (1) be engaging, contain (2) comprehensive and motivating content and (3) features supportive of the quit process, (4) be targeted to pregnancy, (5) feature tailoring of structure and advice, and (6) offer personal contact. A list of additional interview excerpts can be accessed in the supplementary materials [see Additional file 1].

### Theme 1: Engaging ISCI

#### Alternative to traditional support

Participants viewed ISCIs as potentially helpful alternatives to medication, face-to-face or telephone support, and mentioned privacy, convenience, flexibility and constant availability as key advantages of ISCIs. ISCIs were believed to be especially helpful where traditional support is limited, unavailable or not accessible. Other reasons for appreciating ISCIs were personal preferences for quitting without assistance from others, dislike of group therapies, or expecting difficulties or embarrassment when using face-to-face support. For example one participant described how ISCI could overcome some of the challenges that she experienced in relation to accessing support through services: "…you can do it in your own time and you don’t have to go out and see anybody, … because it’s quite an embarrassing thing that it’s not just easy to quit… that you need support, because you have put a day there, but it is difficult, so you can do it [use ISCI] in your own time and yeah, there’s no pressure. So I think that’s quite helpful". (Participant #3, 20 yrs, quit smoking). Other participants, however, felt that ISCIs should be a complementary to other forms of support "I think it needs to be done alongside other support in my experience, so NRT or seeing a Stop Smoking Counselor". (Participant #7, 33 yrs, relapsed and trying to quit).

#### Engaging intervention

Participants believed that ISCI should offer novelty, particularly if it offers long-term support: "I was using it for about five, six, seven weeks and it was the same thing all the time … the same questions over and over again … I think you’d use it a lot more, you’d continue with it a lot more if there was something different every day" (Participant #4, 29 yrs, trying to quit). For example, participants suggested that novelty could be introduced by means of frequently updated or rephrased content and follow-up questions, which would prevent ISCI from becoming "boring" and incentivize re-visits, which participants believed could aid quitting.

Participants also preferred an interactive environment, offering interesting, entertaining and attention-grabbing features that could be explored gradually, and with which they could engage regularly, so facilitating impromptu craving management. This was viewed as particularly important for younger users "… you’re going to have to catch the younger generation, I’m thirty-five and I’m kind of okay with computers and all of that, but the younger lot they’re using smart phones, they really do expect things to be all singing or dancing or they tend to lose interest, so I think cranking up that side of it would be great for the future as well … I think just extend the website and target to young women and put more in it, make it more interactive, more personalized, keep the women engaged and in the loop for as long as possible and I think you’ll greatly increase the chances that she’ll you know, give up" (Participant #12, 35 yrs, quit smoking).

#### Accessible support

Participants preferred an easily accessible intervention, with simple log-in procedures and user-friendly navigation. They believed that ISCIs should also be offered as ‘apps’ on mobile devices, to provide a more practical, convenient and readily-available source of support and additional reminders: "…you could have a really, really great page giving some really, really good advice, but when you’re giving up smoking you need to be constantly reminded of things, because otherwise the cravings take over your thought patterns, so if you have something on you, even if it was just you could read what you’ve read again, just to get you remembering it and to, you know, give you that extra boost and it’s there in your pocket" (Participant #8, 41 yrs, quit smoking).

### Theme 2: Comprehensive and motivating content

#### Reasons to quit

Participants, especially those struggling to initiate the quit attempt, wanted coherent and clear motivational information, including facts on benefits of cessation for baby and mother, and the risks of continued smoking. Some women were actively searching for such information online, and expressed dissatisfaction with the information already available, often seeing it as confusing and contradictory. Moreover, participants expected ISCI to deliver novel, comprehensive and detailed medical information on the harms of smoking to the baby, which they believed to be more motivating, and even capable of ‘shocking them’ into quitting: "…my sort of questions were, you know, what damage does it actually do to the placenta, you know, proper sort of medical facts, not just, ‘Your child might be eight ounces lighter than your next door neighbor’s … it was just all the same old … I haven’t really come across anything that, that scared me enough." (Participant #8, 41 yrs, quit smoking). However, some suggested that the intervention should focus mostly on positive messages and imagery rather than "frightening" content.

#### Quit process and methods

Participants expected comprehensive guidelines for successful quitting, including quit plans and information on the forms of support available to them. Information on likely potential withdrawal symptoms and their intensity and duration was also deemed useful. Participants appreciated comprehensive information on safe and effective medications in pregnancy, and potentially helpful quit strategies, such as self-identifying as non-smokers: "It was about thinking about yourself as a non-smoker rather than as someone that was giving up smoking and that was quite good … I think it just makes you think more positively about it and that it will happen rather than something that you’re trying to do." (Participant #6, 27 yrs, quit smoking).

#### Testimonials

Participants were interested in motivating and informative personal stories and experiences from other pregnant women smokers trying to quit during pregnancy. Some participants wanted access to testimonials of how smoking negatively affected other women, and especially their babies: "I don’t know if there is someone who experienced this impact [of smoking], maybe they can talk about. Like an impact on the baby and it’s more important about the baby" (Participant #2, 26 yr, relapsed). They also felt they would benefit from learning from others’ success stories about how they quit smoking and how ISCIs benefited them: "I think if you put maybe more content from women themselves that have managed to give up while they were pregnant, a variety of different narratives, because I think that for some people that would cut through you know" (Participant #12, 35 yrs, quit smoking).

### Theme 3: Features supporting the quit process

#### Craving management

Participants expressed a strong need for help with craving management. Several MumsQuit features oriented towards coping with craving, including a meditation routine and generic tips and advice, such as adopting physical activity for distraction, were seen as useful even by participants who did not experience strong cravings. Interviewees also believed ISCIs could help them remain abstinent by offering distraction from cravings. One participant described her interaction with MumsQuit website: "I was occupying my mind looking at the different links and looking at all the different tools etc., on there, so I would spend probably about half an hour, 45 minutes on there, by that time my carving had [stopped]" (Participant #5, 25 yrs, quit smoking). Participants therefore expressed positive attitudes towards an ISCI as a potential "treasure trove of resources "with which they could engage until cravings passed, and a need for more features to occupy their hands, such as interactive games.

#### Self-monitoring and feedback

Participants were interested in interactive, self-monitoring tools for tracking progress in quitting and documenting benefits, such as charts and personalized feedback on number of smoke-free days, money saved and health gains: "…a good idea that you can sign up and put down your personal experience and stuff. Because you get to kind of keep a track of your progress and how well you’re doing. Because well you get to see that you’re doing well". (Participant #3, 20 yrs, quit smoking). Diaries for noting experiences and successes, and features offering progress feedback were also reported as providing motivation to continue using the ISCI and remain abstinent: "I was using it every day just to see that you’d given up for another day, which sounds really stupid but it was a really good thing to keep it going" (Participant #5, 25 yrs, quit smoking).

#### Appraisal and encouragement

Many participants reported struggling to quit or remain abstinent, and some reported relapsing to smoking. Most therefore expressed a need for frequent positive support and encouragement, either via the website or e-mails. One interviewee explained how she did not receive the emotional support at home and found the website as the only source of encouragement: "I found it really helpful that you would use positive reinforcement as well. So, even just writing a line that says, ‘Well done, you’re doing really, really well’, sometimes, I’d been finding it so difficult that that would make me cry … I’d be like, "Oh, my God. Thank God someone’s saying ‘Well done’." (Participant #13, 29 yrs, quit smoking). They reported increased motivation and "feeling good about themselves" after receiving positive feedback and appraisal messages from MumsQuit, because this acknowledged their continued abstinence between intervention sessions.

#### E-mail reminders

Participants expressed mixed views towards receiving regular e-mails about smoking cessation as part of ISCIs. Some saw automated e-mails as impersonal, "pushy" and akin to "spam". "The last thing you want is like an automated email system because you can tell that it’s just kind of the usual stuff, you know, that gets sent out to everybody." (Participant #10, 24 yrs, still smoking). Others, however, preferred extensive e-mail support containing additional advice, encouragement, and reminders to re-visit the website or information about content updates. Some reported that regular e-mails helped them to remain engaged with the website, maintain motivation and remind them not to smoke: "…the daily emails and the reminders [were helpful]. Because it reminded me that I’d have to not have one [cigarette] because I’ve got to carry on with that program the next step. "(Participant #4, 29 yrs, trying to quit).

#### Support in relapse

Participants viewed relapse as a common challenge in pregnancy, and emphasized that assistance with failed quit attempts should be offered alongside support for first-time quit attempts within the same ISCI. They expressed strong negative views towards the prospect of an ISCI abruptly terminating support, or encouraging use of traditional smoking cessation support, in response to relapse: "[the intervention] should be seen more as a series of steps that you can back and forward a couple of times … A lot of support for relapse [is needed], I think that that should be part of it if it’s going to be taken seriously …" (Participant #12, 35 yrs, quit smoking). Participants expected ISCIs to provide reassuring, encouraging, and consoling messages after relapse, as well as the possibility of continuing using the intervention, returning to earlier sessions, or resetting the quit date. One participants who relapsed explained: "there should be another chance to try it and set another day for you to quit and actually start again, all over again… I was ready to do [it]." (Participant #2, 26 yrs, relapsed). The participant also felt that the intervention should address the causes of relapse: "…on the website, it asked me ‘why did you go back [to smoking]?’ and mainly because I was really stressed, but there was no help …" (Participant #2, 26 yrs, relapsed).

### Theme 4: Targeting to pregnancy

#### Focus on pregnancy

Participants reported preoccupation with pregnancy "…when you’re pregnant everything you want to know is about pregnancy, it’s about the baby" (Participant #1, 35 yrs, quit smoking). They were therefore enthusiastic about using an ISCI exclusively for pregnant women, as this increased intervention relevance and the importance of using it. They also held positive views towards imagery relating to pregnancy and motherhood, "I loved the positive images that it gave and, if you’re having a really bad craving, and trust me I did, just the picture of a woman holding a flower next to her tummy or something that’s just a really beautiful shot about how pregnancy should be is the type of inspiration that you need…" (Participant #13, 29 yrs, quit smoking). Participants also expressed preferences for relating the feedback reports and appraisals of successes to their pregnancy stage, and particularly their baby’s wellbeing:" the progress is excellent but perhaps link that into the baby as well and how much better health it would be for the baby". (Participant #1, 35 yrs, quit smoking).

#### Non-judgmental advice targeted to pregnancy

Recognizing the importance of quitting smoking in pregnancy and experiencing additional challenges associated with it, participants expected an ISCI to demonstrate understanding of the unique circumstances and difficulties that they face: "…I wanted something that would be tailored for me as a pregnant woman rather than just AN Other party who’re quitting for whatever reason they have" (Participant #12, 35 yrs, quit smoking). They also wanted be to approach in a friendly, positive, "non-judgmental" and "non-preachy" manner, and to receive advice that was not only novel, but also specific to pregnancy, instead of ‘generic’ information that is already available online for all smokers. This was also provided as a reasons for joining the present study: "I hoped that it might be better than, you know, more generic stuff that one finds because it’s, you know, a new study and, you know, particularly tailored to women in pregnancy which I hadn’t found before" (Participant #7, 33 yrs, relapsed, trying to quit).

### Theme 5: Structure and advice tailoring

#### Flexible intervention structure

Participants valued flexibility in setting a quit date, including an option to set it for the day of joining the ISCI without needing to complete pre-quit sessions. This was suggested as particularly important for women who would access the intervention after having already initiated a quit attempt, which was also the case of one participant: Although some women appreciated a guided, step-by-step ISCI structure, others were discouraged by restricted navigation of the website content and instead preferred more freedom in accessing all of the ISCI information and tools. "…I think … if you want to sit there and go through the whole thing and then revisit it I think you should be able to do that. I think you’ll find that people will join up to it if they’ve already started quitting but are having a hard time, they probably need to get to [session] two or three fairly quickly". (Participant #6, 27 yrs, quit smoking). Differences were also found between participants’ needs for the amount of information as well as the intensity, pace and duration of support offered. For example, some participants were dissatisfied with the 4-week program of decreasing intensity offered by MumsQuit, and expected a more lasting source of help.

#### Smoking cessation medications

Women were interested in learning about smoking cessation medications, but differed in attitudes towards using these in pregnancy. Those who did not want to use NRT or stopped using it during the trial expressed dissatisfaction with being encouraged to use it, and some worried that the intervention may be an undeclared commercial website promoting pharmaceuticals. Women who tried using NRT as part of their quit attempt expected more support tailored to their experiences with medication used, including changes in NRT used, as well as advice on when and how to use NRT best: "Maybe ask a few more questions on how you’re feeling and things like that [regarding NRT]. I think is the product working? Are they effective, are they giving you side-effects? You know, is it feasible for your way of life? So it encourages you to go to something else". (Participant #4, 29 yrs, trying to quit).

### Theme 6: Personal contact

#### Peer-to-peer communication

Participants felt encouraged to know that other women were using the same ISCI to quit smoking. However, they also expressed strong preferences for ISCI offering communication with other pregnant women wanting to quit smoking (e.g. a discussion forum), viewing it as a very valuable source of advice and support. They believed that it could enable them to share concerns and "spur one another [on] in difficult moments", such as when experiencing cravings: "…if you’re having a bad moment you really want to smoke at that point it was sort of like really right at this moment I need to speak to someone… and I’d go on the website now, without having to go to any trouble of phoning someone or anything like that, I’d just like to go and chat with somebody about it "(Participant #1, 35 yrs, quit smoking). However, participants stressed that the peer-to-peer facilities should only be accessible to pregnant smokers wanting to quit: "it’s important that they would be just for members that are trying to do the same thing. Yeah, you don’t want people on there that just are anti-smoking in pregnancy and think that they can preach and upset everybody because that wouldn’t help". (Participant #6, 27 yrs, quit smoking). Therefore, participants expected the discussion forum to offer an empathetic social environment, and to protect users from potentially stigmatizing, upsetting and judgmental comments from non-smokers.

#### Personal support from experts

Some participants expected to receive support from experts, such as stop smoking advisors or ISCI developers, and were reluctant to use an ISCI not offering such contact. One participant explained that lack of personal contact led her to stop using MumsQuit: "I thought I don’t know if it’s enough support for me, …, I need more than online support, I think I need more like people, somebody to contact…" (Participant #11, 40 yrs, trying to quit). Participants emphasized that personal support would increase attractiveness of ISCIs, and that a brief e-mail exchange or single telephone call with an expert could increase their success, especially when struggling to maintain abstinence. As one participant explained: "Even if it was just to take two minutes just to phone me would be amazing. It would make me feel more looked after, more cared for …that I mattered, that my child mattered and that what I was doing was really important. … I needed someone just to go, ‘Are you okay? You’re doing really well. Get on with it, keep going’. That would be great. Yeah, it would make me feel empowered". (Participant #13, 29 yrs, quit smoking).

### Participants’ underlying core needs

Through a systematic analysis of the participants’ preferences for specific ISCI features outlined above and the context within which they emerged throughout the interview we identified five core needs behind participants’ accounts. These were: to be well-informed about smoking and cessation in pregnancy; to be understood and appropriately addressed as a pregnant woman attempting to quit; to be motivated and committed to quitting; to remain engaged with the ISCI; and to receive ongoing assistance with remaining abstinent. Table 1 presents a synthesis matrix linking these two sets of findings: the preferences for specific ISCI features (presented in rows) and the core needs that seem to have been underlying participants’ preferences for these features (presented in columns). For example, participants’ preferences for a discussion forum seemed to have been motivated by a need to gain information, be in an understanding environment, receive assistance with cravings, and be provided with a reason to regularly engage with the ISCI.

**Table 1:** **ISCI feature and the core needs of pregnant smokers that may be underlying them**

ISCI features and components:	Underlying core needs:				
	To be well-informed	To be understood	To be motivated	To be engaged with ISCI	To be assisted in abstinence
**Engaging ISCI**					
**Engaging intervention**					
Frequently updated and novel advice and information	●			●	
Numerous interactive resources, games and discussion forums				●	●
**Accessible support**					
User-friendly navigation and log-in procedures				●	
Additional modes of delivery (e.g. "apps" for mobile devices)				●	●
**Comprehensive and motivating content**					
**Reasons to quit**					
Benefits of quitting for the fetus and the mother	●		●		
Risk of continued smoking to the fetus and pregnancy	●		●		
Framing the information positively		●	●		
Comprehensive, detailed and novel information	●		●	●	
**Quit process and methods**					
Planning a quit in pregnancy	●				
Quit methods, support and medication for pregnant women	●				
Withdrawal symptoms, and their expected duration, intensity	●				
**Testimonials**					
Women’s experiences of the impact of smoking on babies	●		●		
Women’s stories of how they quit successfully	●		●		
Experiences of women who quit smoking using ISCI	●			●	
**Features supporting quit**					
**Craving management**					
Relaxation activities, e.g. meditation					●
Tips and advice, also for those reporting low cravings	●				●
Environment rich in resources aiding distraction, e.g. games				●	●
**Monitoring and feedback**					
Interactive progress reports on benefits of quitting	●		●	●	
Diaries to record progress and successes			●	●	
**Appraisal and encouragement**					
Frequent encouragement to continue with the quit			●		
Frequent appraisal of successful abstinence			●	●	
**E-mail reminders**					
Newsletters with information, advice and encouragement	●				
Prompts to re-visit the website; information on updates				●	●
**Support in relapse**					
Consoling and reassuring messages following relapse		●	●		
Option to re-visiting earlier modules and re-set quit date				●	
Providing support and advice addressing the causes of relapse		●			
**Targeting to pregnancy**					
**Focus on pregnancy**					
Use of imagery related to pregnancy and motherhood			●	●	●
Linking progress and advice to baby’s health and pregnancy			●	●	
**Non-judgmental advice targeted to quitting in pregnancy**					
Advice on quitting methods during pregnancy	●				
Advice addressing additional challenges of quitting in pregnancy		●			
Non-judgmental, non-preachy, and understanding approach		●	●	●	
**Structure and advice tailoring**					
**Flexible intervention structure**					
Accounting for the needs and preferences for pace of support				●	
Tailoring to stage and experience with quit at the start		●		●	
Flexibility in setting up the quit date, e.g. on the day of signing up				●	
**Stop smoking medication**					
Advice tailored to attitudes towards medication use		●			
Extended support and advice for women using medication	●				
**Personal support**					
Discussion forums only for other pregnant women trying to quit	●	●		●	●
Proactive or reactive support from experts (calls, chat or e-mail)		●	●	●	●

## Discussion

The present interview study with participants using a prototype ISCI especially targeted to pregnancy has lend us first insights on what features and components may be attractive and potentially helpful to this population. Pregnant women seeking online support with smoking cessation may view ISCIs as helpful alternatives to traditional cessation support because of the privacy, convenience, flexibility and constant availability, particularly when they face barriers to engage with established cessation services. However, the results also suggest that ISCI might offer valuable assistance in addition to the support that women may be already accessing. An engaging, highly accessible and interactive ISCI that incentivizes re-visits and offers non-judgmental support tailored to the individual circumstances of users may be attractive to pregnant smokers who are both attempting to quit as well as trying to remain abstinent. Conversely, repetitive, too generic and prescriptive advice may cause dissatisfaction and disengagement from ISCI use.

Pregnant smokers interested in using ISCI may also value comprehensive targeting of ISCIs to pregnancy, from the smoking cessation information and advice offered, through feedback on progress, to the imagery used. This study supports past findings showing that targeting may increase satisfaction, perceived relevance and effectiveness of advice [39–41]. We also found that women may differ in their preferences for accessing information on the negative health outcomes of smoking, and such content could be therefore made optional for women to access. Future quantitative studies should explore the impact of intensive targeting to pregnancy to uptake, usage and effectiveness.

Several ISCI components were particularly desirable to participants. Provision of extensive post-relapse support was viewed as an integral part of the intervention, and might be especially important given the high relapse rates among pregnant smokers (around 23%) [42]. Identifying and distinguishing lapses from relapse [42, 43], as well as their appropriate management in this population using ISCI warrant further research. Additionally, offering a personal support from an expert in smoking cessation was also viewed favorably, and indeed has been shown elsewhere to improve the effectiveness and usability of ISCI [44]. Personal support has been offered in a number of other internet-based smoking cessation interventions, and involved an online chat with cessation ‘experts’ or provision of a telephone line in case of strong cravings [22] as well as more proactive telephone support from counselors [45]. Future studies of ISCIs for pregnant women may also assess inclusion of personal support from midwives trained in smoking cessation.

Online communication with other pregnant women trying to quit were also viewed as useful, particularly among those trying to remain abstinent. This could provide a viable alternative to traditional ‘buddy-up’ interventions that are often difficult to implement in practice [46]. However, while online social support may enhance users’ coping skills, motivation, website use [46, 47], and increase abstinence rates [48], it might also require external moderation to prevent transmission of misinformation [49]. Participants also reported being motivated by the perceived social presence of other women accessing the website. This has been shown to be important for behavior change [50], but its effectiveness has not been investigated in ISCI trials.

The sample spontaneously identified as useful many BCTs that have previously been shown to form part of effective smoking cessation interventions in pregnancy, including provision of information on consequences of smoking and smoking cessation, and rewards contingent on successfully stopping smoking, as well as strengthening ex-smoker identity [51]. Strengthening a non-smoker identity in pregnant women or mother offers a relatively novel and potentially useful BCT for use within an ISCI, particularly given participants’ positive reception of imagery appealing to these identities within the ISCI. This might also involve encouraging women to think of themselves as a non-smoking, health-conscious mother-to-be who cares for her own health and the baby’s health and wellbeing, and therefore does not smoke. However, effectiveness and acceptability of these components require further research.

Overall satisfaction with the ISCI may further affect women’s engagement with the program and, indirectly, its effectiveness [52]. Our findings suggest that the attractiveness and relevance of a minimally tailored ISCI to pregnant women might be increased by more comprehensive tailoring of the intervention to pregnancy stage, stage of quit at the start of the program, and attitudes towards smoking cessation medication [53]. Furthermore, it was reported that e-mails may increase engagement with online interventions [54]; however, this study suggest that individual preferences for the intensity of e-mail communications and personal support should be taken into account. Importantly, provision of additional support on mobile devises, e.g., through text-messaging or smartphone applications may further increase convenience, appeal and intervention engagement among this population, particularly when the advice would be highly personalized, although it may present challenges, including risk of message devaluation [12].

A highly structured ISCI that provides support only at pre-determined intervals of decreasing frequency may not be acceptable for all women. Previous research has found that having unrestricted access to all intervention content increased satisfaction and predicted smoking cessation, yet also had a negative impact on gains in knowledge and website use [48, 55]. Future studies should assess whether satisfaction and intervention effectiveness improve when more intense and prolonged support is further tailored to participants’ timing needs.

Findings suggest that pregnant women’s preferences regarding ISCI may stem from their need to be informed, understood, encouraged, and engaged with the ISCI, while also being assisted to remain abstinent. ISCI attractiveness may therefore depend on addressing all of these five domains. Although these observations are in line with past findings on web-based behavior change interventions [54, 56], the potential for these preferences to determine intervention use and effectiveness among pregnant smokers offers hypotheses for future quantitative studies. Future work should also evaluate whether ISCIs can sufficiently address these needs. An implicit assumption of our study was that pregnant women have relatively homogeneous ISCI preferences and needs. Future qualitative and quantitative work might investigate whether personal pregnancy circumstances, such as parity or medical complications, might affect views towards ISCIs or uptake rates and, if so, whether ISCIs might be tailored to increase personal relevance and uptake.

Finally, although ISCIs may be particularly attractive to women who are not accessing other support, the present study suggests that at least some pregnant smokers might be interested in using ISCIs alongside traditional interventions. Smoking cessation experts and midwives working with pregnant smokers were recently shown to view digital interventions, including ISCIS, as potentially helpful aids to support cessation treatment for this population [18]. Moreover, computerized interventions for perinatal drug use offered within a hospital setting were also acceptable to post-partum women [57, 58]. There is a therefore a need to examine how ISCIs might best assist pregnant smokers in the context of other cessation support available to them, and particularly, how such support could be integrated with the existing stop smoking services, antenatal clinics, or health visitors and care workers, perhaps through a referral system to use the ISCI, and through monitoring of its use and outcomes. Additionally, economic evaluations of the long-term sustainability of ISCIs should be conducted to identify and contextualize the cost burden in health systems that face increasing health care costs.

Study limitations should be acknowledged. The sample was predominantly composed of white British and married women, which limits the generalizability of experiences identified to other pregnant smokers. However, the aim of the present study was not to generate generalizable findings, but rather to elucidate various needs and preferences that can be investigated in quantitative studies. Nonetheless, further work is needed to explore whether, for example, ethnic minority or adolescent pregnant smokers hold preferences not captured among our sample. The small sample recruited also reflects the challenges of engaging pregnant smokers in research see also [59, 60], but theoretical saturation appeared to be reached, with no new themes emerging in the last three interviews [61]. Although the sample included a similar number of smokers and non-smokers, it is possible that some trial participants who refused to be interviewed did so because of feelings of shame and embarrassment associated with continued smoking. Additionally, some participants who were already non-smokers at the time of the interview may hold different views to those who continued smoking.

Finally, an important limitation of the present study is that interviews were conducted only with women allocated to use the MumsQuit intervention, and not those allocated to a control website. It is therefore possible that some of the views expressed were specific to MumsQuit rather than to ISCIs more generally. Documenting the views of the control group may have allowed for more reliable identification of preferences generalizable across a range of ISCIs. However, the primary aim of the project from which this study was drawn was to understand the acceptability of ISCIs in relation to structured interventions such as MumsQuit, and so views towards the control site were not sought. Additionally, while interviewees were not explicitly asked to access and test other websites, several participants nonetheless reported having used other digital resources, and they were encouraged to share their experiences and views on these, which has been incorporated into the results. This increases our confidence that findings capture needs and preferences towards ISCIs, and not solely the MumsQuit intervention. However, future intervention development studies might usefully seek to capture responses to a range of interventions and potential features to better contextualize the views expressed towards the focal intervention. Notwithstanding these limitations, this study reveals the various needs and preferences around ISCIs, as expressed by women who already have experience of an intervention that was minimally targeted to pregnancy and highly-structured (i.e. MumsQuit), and who represented a range of views and levels of engagement with the program.

## Conclusions

An engaging, structured ISCI highly tailored and targeted to pregnancy, and additionally combined with personal contact may be attractive to pregnant smokers seeking help online. Quantitative and experimental studies using larger samples in different countries and settings are required to determine whether interventions addressing the identified needs and preferences would be effective among pregnant smokers. There is also a need to conduct population-based surveys to quantify the demand and accessibility of ISCIs among this group.

## Electronic supplementary material

Additional file 1: **"The needs and preferences of pregnant smokers regarding tailored Internet-based Smoking Cessation Interventions: a qualitative interview study.** Additional interview excerpts" provides a list of relevant additional quotes from participants, which are arranged and labeled in the same manner as the results present in the main manuscript. (DOC 112 KB)

## Abbreviations

ISCI: Internet-based smoking cessation intervention
NRT: Nicotine replacement therapy
BCT: Behavior change technique.
