# Supplementary material for: The needs and preferences of pregnant smokers regarding tailored Internet-based smoking cessation interventions: a qualitative interview study
Source: BMC Public Health. 2014 Oct 14;14:1070. doi: 10.1186/1471-2458-14-1070 (PMC4209063; doi:10.1186/1471-2458-14-1070)
Supplement: Supplementary file 1 — Additional file 1: "The needs and preferences of pregnant smokers regarding tailored Internet-based Smoking Cessation Interventions: a qualitative interview study. Additional interview excerpts" provides a list of relevant additional quotes from participants, which are arranged and labeled in the same manner as the results present in the main manuscript. (DOC 112 KB) [file 12889_2014_7171_MOESM1_ESM.doc]

**The needs and preferences of pregnant smokers regarding tailored Internet-based Smoking Cessation Interventions (ISCIs): a qualitative interview study.**

**Additional File 1: Additional Interview excerpts.**

Aleksandra Herbec^1^, Emma Beard^2^, Jamie Brown^2^, Benjamin Gardner^1^, Ildiko Tombor, ^1^ & Robert West.^1,3^

^1^ Cancer Research UK Health Behaviour Research Centre, Department of Epidemiology and Public Health, University College London, Gower Street, London WC1E 6BT, UK

^2^ Clinical, Educational and Health Psychology, University College London, 1-19 Torrington Place, London WC1E 7HB, UK

^3^ National Centre for Smoking Cessation and Training, London, UK

**Corresponding author:** Aleksandra Herbec, M.Sc.

Health Behaviour Research Centre.

Department of Epidemiology and Public Health,

Rm 215

University College London

Gower Street

WC1E 6BT, UK

E-mail: aaherbec@gmail.com

| **Theme 1: Engaging ISCI** | | | | |
| --- | --- | --- | --- | --- |
| **Higher order subthemes** | | **Lower order subthemes** | | **Example interview excerpts** |
| **1.1. Alternative to traditional support** | | **1.1.1 Advantages of online support** | | “….somewhere where you can, you don’t need to pick up the phone and I think, particularly with the way that that’s, that’s the way the world is going now, it’s very much technology based, I just think the idea of having a website that ladies could access 24/7 is a really good idea..” (Participant #1, 35yrs, quit smoking)  “I thought, “great” [convenient] because to join one of the sort of groups you know, the GP’s practice that you know, or that kind of thing, run would mean sort of committing to a particular day and date and time whereas this I could do at a time that suited me, so it was the convenience I think that appealed, also the privacy I think, I think that helped. (Participant #12, 35yrs, quit smoking) |
|  | |  | | “The thing I loved about your website is you would help me every single day. There is no way that a NHS nurse would help me every day and say something positive every single day. So, that’s not a proper support, is it?” (Participant #13, 29yrs, quit smoking) |
|  | | **1.1.2. Barriers to accessing traditional support** | | “...to be able to access that, you know, at times when for instance you can’t go to a support group or you know you’re at work and you don’t have NRT on you or something like that…” (Participant #7, 33, relapsed and trying to quit)  “I think you should go nationwide and I think you could help a lot of people. I really think you should be part of the NHS. I think you should offer your services out to help people, because sometimes when the NHS does fall down, well, maybe you. guys could help […] you could do locations where people maybe. couldn’t get to the doctor or couldn’t go to a help session or whatever...”(Participant #13, 29yrs, quit smoking) |
|  | |  | | “Is the way forward, particularly when you’re pregnant if you’re having difficulty giving up smoking, it can be quite embarrassing, at times humiliating, not that anyone’s trying to do that to you but it can feel quite, you don’t want to be outside smoking a cigarette looking pregnant, you also don’t want to be necessarily going in and out of a GP smoking clinic with AN Other Person….(Participant #12, 35yrs, quit smoking) |
|  | | **1.1.3. Combined support** | | “…I think it needs to be done alongside other support in my experience, so NRT or seeing a Stop Smoking Counsellor.” (Participant #7, 33, relapsed and trying to quit) |
| **1.2. Engaging intervention** | **1.2.1. Novelty** | | “Or have something that people can go on and there’s something different for them each day, like a, you know, one month programme that every day they sort of work through and it gives them little sort of supportive tips or, um, sort of challenges or that kind of thing, just something to keep the interest up and [...] keep supporting and maintaining people’s motivation to stop.” (Participant #7, 33, relapsed and trying to quit)  “Maybe just, not all the time, but maybe every other week, every other day maybe put a little bit more information on there about the effects because if you read it every day it’s just like a broken record. If you put a note every other day just to remind somebody, you know, if you carry on smoking you’re going to do this” (Participant #4, 29yrs, trying to quit) | |
|  | **1.2.2 Interactive and Rich environment** | | “…your website is really good because it’s almost like a treasure trove of things that you can find and you can explore it when you’re ready” (Participant #13, 29yrs, quit smoking)  “...it’s better to have more I think, more choices or more options and more things to do to engage with the website than less” (Participant #12, 35yrs, quit smoking)  “…the interaction (with others) thing [...] I would have liked to have been more, [...] ‘cos then you could have spent an awful lot more time, particularly when you were having a bad moment you could have more of it…. I’d go on the website now, without having to go to any trouble of phoning someone or anything like that, I’d just like to go and chat with somebody about it.” (Participant #1, 35yrs, quit smoking) | |
| **1.3. Accessible support** | **1.3.1. Navigation** | | “Um, you know if you do log in to do something to then have to, once that’s finished to log back in again is just a bit annoying,…I just get bored easily…So just sort of make you be able to click through onto the next bit rather than almost having to start your session all over again.” (Participant #6, 27yrs, quit smoking)”  “...not too many, um, not too much on one page for starters, so you know, really, um, you need to follow links, I don’t mind if I have to click ten times to get to where I need to go but they need to be easy to actually find what you’re looking for on the site.” (Participant #7, 33, relapsed and trying to quit) | |
|  | **1.3.2. Mobile devices** | | “I think it’s really important that it’s accessible from mobile phones and things like that because a) that’s how a lot of people choose to browse when they’ve got the choice but b) that’s how a lot of people have to browse if they haven’t got the choice. [using iPad] I think it’s just convenience really. ” (Participant #6, 27yrs, quit smoking)  “..you’re going to have to catch the younger generation, I’m thirty-five and I’m kind of okay with computers and all of that, but the younger lot they’re using smart phones, they really do expect things to be all singing or dancing or they tend to lose interest, so I think cranking up that side of it would be great for the future as well.” (Participant #12, 35yrs, quit smoking) | |
| **Theme 2: Comprehensive and motivating content** | | | | |
| **2.1. Reasons to Quit** | **2.1.1. Views on the information available online** | | | “... There’s so much contrasting information out there, erm... You know, my sort of questions were, you know, what damage does it actually do to the placenta, you know, proper sort of medical facts, not just, “Your child might be eight ounces lighter than your next door neighbour’s.” It was all a bit washy. There was nothing that really shocked me into thinking, “Oh God!” it was just all the same old, you know, low birth weight, might be born a bit earlier. I haven’t really come across anything that, that scared me enough...” (Participant #8, 41yrs, quit smoking)  “I think all the stuff that tends to be on sort of specific pregnancy websites or forums tends to be quite preachy…” (Participant #6, 27yrs, quit smoking)  “I think when I was looking at information when I was wanting to stop it just felt really depressing that every kind of, you know everything I looked at was just telling you how terrible it was that you were still smoking, you know (Participant #10, 24yrs, still smoking) |
|  | **2.1.2. Benefits of quitting** | | | “…like specific information about the, you know, health benefits, like over, you know, if you stop for one week this is, you know, what’s happening and, you know, feeling in your body. (Participant #7, 33, relapsed and trying to quit)  “…[add information on] how they’re going to feel after they quit, so they don’t know how they’re going to feel, like I knew it’s a good feeling, it’s not a bad feeling and they’re going through quickly and then they’re going to be feeling bad, feeling healthier and they can breathe more easily and they can do… they can walk and they can do more stuff. And also the release from the stress of wanting to have a cigarette or thinking about it and all this stuff… […] this is very important because you forget how you feel without smoking […] it would be a motivator.”(Participant #2, 26yrs, relapse and trying to quit) |
|  | **2.1.3. Novel information on risks of Smoking – motivating information** | | | “...the facts on smoking and see the effects it’s having on the baby which is quite helpful because you do feel guilty for smoking [...] it kind of makes you realise what you’re doing, so that’s it I think, it spurs you on “(Participant #3, 20yrs, quit smoking)  “I was putting a pregnancy phrase in [Google search], because I’ve tried all the other “stop smoking” stuff before, you know, so... and I thought maybe this might be a bit of shock tactic somewhere for me (Participant #8, 41yrs, quit smoking).  ‘Maybe put more details on it about how the smoke affects the fetus, the unborn baby, because a lot of the time if you were to say well the cigarette smoke does this to your baby, that shocks a lot of people into stopping maybe put a little bit more on the effects of smoking on a normal baby (Participant #4, 29yrs, trying to quit) |
|  | **2.1.4. Positive message framing** | | | 1. “So, you could add that to it as a separate page [with risks]. However, I don’t think you should put that on the first page you see as you’re trying to give up, because it’s too frightening (laughs). Use the positive images, you know […] You don’t need photos of, you know, a damaged baby and all this kind of stuff. So, actually, you did the right thing, you did positive images, and when I was really angry or really upset or really frustrated, that was really helpful.?” (Participant #13, 29yrs, quit smoking) |
| **2.2. Quit process and methods** | **2.2.1. Quit process and methods** | | | ‘I had a look kind of online at you know some information and stuff and research, […] just information about what I could expect if I quit smoking, you know, kind of um, side-effects, […] you know things that kind of, things to help me get through it.’ (Participant #10, 24yrs, still smoking)  “... I think if you was to put on all the [NRT] options that are available and what the product is because a lot of people don’t know about certain products so maybe if you put on there that “these products will help you” put a bit on that on there.” (Participant #7, 33, relapsed and trying to quit) |
|  |
|  | **2.2.2. Non-smoker identity** | | | 1. “It was about thinking about yourself as a non-smoker rather than as someone that was giving up smoking and that was quite good [..] ‘giving up’ implies something that you’re still trying to do, non-smoker is like someone that’s made that decision. I think it just makes you think more positively about it and that it will happen rather than something that you’re trying to do.” (Participant #6, 27yrs, quit smoking) |
| **2.3. Testimonials** | **2.3.1. Impact of smoking** | | | “..that website [another website that the participant accessed] was really helpful […] also, because some people who have cancer were writing their diaries on the website, [...] I found it helpful because they were writing about it every day and how they’re feeling and how they regret that they didn’t quit. And, actually, one of the ladies, she passed away whilst she was writing her diaries on the website. [...]it’s a very important thing, you can relate to people who were in the same situation.” (Participant #2, 26yrs, relapse and trying to quit)” |
|  | **2.3.2. Strategies that work** | | | “I think if you put maybe more content from women themselves that have managed to give up while they were pregnant, a variety of different narratives, because I think that for some people that would cut through you know, some people don’t engage very well with things that are either suggesting things that they do or being educational, so I think if there was some narratives that might help some folk” (Participant #12, 35yrs, quit smoking)  “... that’s what I’m doing also, I like looking what other people have wrote and what they’ve, how they’ve dealt with it. [I find it helpful]” (Participant #9, 32yrs, trying to quit) |
|  | **2.3.3. Benefits of using ISCI** | | | “...you like positive things to read and see what other people have done and how it’s helped them. Yeah, that would, if I read other stories from other pregnant women that had used the site and had stopped it would make me feel a lot more inclined to use it.” (Participant #11, 40yrs, trying to quit)  “I was a little bit apprehensive, obviously I knew it being on the NHS website it had to be a safe website but I was a little apprehensive at first as to, as to the safety of the website… I didn’t feel like I could trust it because I wasn’t recommended it by anyone…” (Participant #5, 25yrs, quit smoking) |
| **Theme 3: Features supporting the quit process** | | | | |
| **3.1. Craving management** | **3.1.1. Meditation and relaxation** | | | 1. “The meditation that was on there that you use the link for was absolutely brilliant. Absolutely brilliant. Beautiful, in fact, and really, really helpful. I wish there was a direct link to the meditation for those days because meditation would have been so helpful on day three and four […]? I really liked the way you said that if you have a craving, it’s only going to take three minutes or something. Because there was sometimes when I was having a craving and it would feel like a lifetime. Actually, three minutes is okay if you time it. So, I just put a digital timer next to my bed and then try and breathe.” (Participant #13, 29yrs, quit smoking) |
|  | **3.1.2. Tips and Advice** | | | “Um, but for me really it’s just the advice, the advice is helpful […] if there’s anything different I write that down and then I try and use that if I get a bit stressed or if I’m thinking like, you know, I want a fag (laughs).” (Participant #9, 32yrs, trying to quit)  “Um, that kind of thing, also here’s, you know, like really healthy snacks you can make today to, you know, if you’re having cravings.” (Participant #7, 33, relapsed and trying to quit)  “…it came up with some really good ideas some days, you know, like to do exercise and take your mind off things so it did have some really good, um, good suggestions.. [but] once you’d actually been on once it just didn’t seem to really, it just asked you every day how your cravings were but I suppose it’s ‘cos my cravings were okay, I don’t know whether it was ‘cos I was feeling was it okay anyway... it didn’t really bring anything up” (Participant #1, 35yrs, quit smoking) |
|  | **3.1.3. Distraction** | | | “…maybe just have a couple of interactive things like quizzes on how much a person might have learnt, or games which are also based on information that helps to do with stopping smoking, or specifically with pregnant women to stop smoking you know, the kind of things that, say if you wake up in the middle of the night and you know, you’re finding that you’re having cravings for a cigarette, a real urge to go and have a cigarette, and you think, “oh gosh”, you can go on to the website and you’ve got a couple of things that you can do that aren’t necessarily a session but they’re ways that you feel like you can engage with it, with the website and with the process, that would be useful, educational I suppose.” (Participant #12, 35yrs, quit smoking)  “I think they should be like a little bit more, maybe put a little game on there that when you do need a cigarette you’ve got a little game to keep your fingers occupied. Something like that, just something more hands on instead of just constantly reading things.” (Participant #4, 29yrs, trying to quit) |
| **3.2. Self-Monitoring and Feedback** | **3.2.1. Progress charts, timelines, and diaries** | | | “However, it would be really great if I could click on to a timeline and look myself about the progress that I was making.” (Participant #13, 29yrs, quit smoking)  “To be honest I go more on the Stay Quit [website]. because that’s the one where you can like write down and stuff. Um, what it’s just so you can go back and like I say if I’ve had a stressful day where. I’ve really craved a fag, um, but I’ve got through that day I’ll have broke down, um (sighs), you know, how I coped with, um, what I did to get me over that specific craving” (Participant #9, 32yrs, trying to quit) |
| **3.3. Appraisal and**  **Encouragement** |  | | | “Um, something... um, I don’t think you did this, um, like daily email, you know, with some sort of, um, positive supportive, you know, information or message, just as a, you know [...] if they’re having a particular hard day then it’s just an extra little push for them. I think something like that would be really really good.” (Participant #7, 33, relapsed and trying to quit)   1. “… I think I was just hoping that the... it would provide just a bit of reassurance and a bit of information, and you know just positiveness, that’s not even a word, but oh I don’t know, it was difficult in the start. I don’t know, I think just kind of, I feel that you know, yes you can actually do this…” (Participant #10, 24yrs, still smoking)   “… it would come up with obviously a series of questions regarding if you’d had a cigarette since the last time that you logged in, which I really liked because even, because obviously my answer was always ‘no, I haven’t had a cigarette’, […] it was sort of an appraisal and really made you feel good about yourself, yeah. (Participant #5, 25yrs, quit smoking) |
| **3.4. E-mail reminders** | **3.4.1. Reminders about quitting** | | | “…you don’t want it too pushy, I think that was fine, yeah, because sometimes if it’s too much it would just put you, you wouldn’t even read it, you would just ignore it you know, it would turn in to like spam, you would be like, “well they’re just annoying me now”.” (Participant #11, 40yrs, trying to quit)  “the last thing you want is like an automated email system because you can tell that it’s just kind of the usual stuff, you know, that gets sent out to everybody and sometimes it’s nice just to have that personal touch in it.” (Participant #10, 24yrs, still smoking)  “….useful ‘cos it, every day it sort of gave you reminder that you were giving up.” (Participant #1, 35yrs, quit smoking) |
|  | **3.4.2. Reminders to revisit the programme** | | | “… I logged on every single time it told me to via email... but not since, since the study finished.” (Participant #1, 35yrs, quit smoking)  “Sending daily e-mails would be helpful I think […] with come and look at the site and suggestions for other areas of the site to look at, you know, so that if people want to they can spend, you know, more time engaging with the site” …unless there’s frequent reminders that they’re, you know, engaged with this website and with this approach then, you know, I think it’s easy sometimes to just to forget to let life take over and to struggle.” (Participant #7, 33, relapsed and trying to quit) |
| **3.5. Support in relapse** | **3.5.1. Consoling messages** | | | “I think it should be about keeping going rather than seeing it as a one track lane kind of thing where the minute they mess up and smoke, because there could be a lot of reasons why you know…..There should be something written in by these people for the pregnant women if she has relapsed, something that helps,. something that actually, I don’t know, some information, something positive in there you know, rather than it just being, “right, okay, we’ve washed our hands of you, go to your GP”, (Participant #12, 35yrs, quit smoking) |
|  | **3.5.2. Second chance** | | | “I think that there has to be some sort of leniency or approach that is a bit less sudden I think maybe something else should be maybe put in there at that point, that allows you to either go back into core sessions and pick it up from there or something a little less sudden, (Participant #12, 35yrs, quit smoking) |
|  | **3.5.3. Causes of relapse** | | | “…the options are basically, have you smoked, yes or no”, there wasn’t any meaning or context behind it, […] and then you immediately just get a message saying, “well, bye then, go and see your GP”, and that’s the end of it”. (Participant #12, 35yrs, quit smoking) |
| **Theme 4: Targeting to pregnancy** | | | | |
| **4.1. Focus on pregnancy** | **4.1.1. Importance** | | | 1. “it was useful, the idea was useful and that it was just for mums, so you feel more […] you’re more targeted, you feel it’s important to do it, because they are just for mums” (Participant #2, 26yrs, relapse and trying to quit) |
|  | **4.1.2. Linking progress to baby’s wellbeing**  **and pregnancy stage** | | | “And also maybe combining that with also, you know, if you’re around the child’s development so that people are also like, you know, get really connected to like why it’s so important to stop, you know, and relating it to the pregnancy.”(Participant #7, 33, relapsed and trying to quit)   1. “..have it a little bit more related to the pregnancy, and maybe that could be included in, when you sort of sign up, I can’t remember if there was anything that asked you how far in your pregnancy you were, so maybe some questions related to that [or] for instance when you get the, when you first log on and you’re asked if you’ve had a cigarette and you say, "no, I haven’t, and they say, “oh, well done and this is your second day that you haven’t had a cigarette”, maybe then have something related to the beneficial, the benefits of giving up for your unborn baby, and have it a little bit more related to the pregnancy” (Participant #5, 25yrs, quit smoking) |
| **4.2. Non-judgmental advice targeted to pregnancy** | **4.2.1 Addressing the unique circumstances** | | | “I think that’s good that it’s just for pregnant smokers to be honest because that’s what pregnant woman are looking for init, if you go on the NHS that’s to everyone, but when you’re pregnant and you’re speaking to other pregnant women that are going through exactly the same then I think that’s better, a lot better actually….’cos if you go on the NHS then it’s a bloke that’s quit, he’s not gonna know what you’re feeling when you’re pregnant” (Participant #9, 32yrs, trying to quit)  “Um, I hoped that it might be, um, better than, you know, more generic, um, stuff that one finds because it’s, you know, a new study and, you know, particularly tailored to women in pregnancy which I hadn’t found before.” (Participant #7, 33, relapsed and trying to quit) “ |
|  | **4.2.2. Non-judgmental and targeted approach** | | | “I found it encouraging that other people were going through it and that there was support out there for quitting smoking, that it weren’t just expected of you, it was people understand that it’s difficult.” (Participant #3, 20yrs, quit smoking)  “Um, well like I said it was just nice to know that there was support out there just aimed at pregnant people, it [MumsQuit] kind of felt like a no judgment thing like it was […] it was informative but it wasn’t ‘naggy’ […]. Um, it wasn’t preachy, I think it accepted that people knew they should be giving up smoking, um, and it didn’t sort of preach about it.” (Participant #6, 27yrs, quit smoking) |
| 1. **Theme 5: Structure and advice tailoring** | | | | |
| **5.1. Structure and tailoring** | **5.1.1. Quit date** | | | “When I first looked at it and it gave a due date, I was quite disappointed because I didn’t want to give a due date to stop smoking if it was in a few days. I wanted to give up smoking that day, and it didn’t allow you to do that. So, I had to lie… So, the only criticism I’d give is you should allow people to make their own decision about when they give up smoking. If they want to give up that day, then they should.” (Participant #13, 29yrs, quit smoking). |
|  | **5.1.2. Preferences for intervention structure** | | | “I found it very user-friendly, you know, the sort of step-by-step thing,” (Participant #7, 33, relapsed and trying to quit)  “…I think you have to do like one module and then wait for a next module to be sent. And that was where I lost interest […] I think you need to be able to do it in your own time, if you want to sit there and go through the whole thing and then revisit it I think you should be able to do that” (Participant #6, 27yrs, quit smoking) |
|  | **5.1.3. Needs for support** | | | “...more sessions, more bits and bobs to it, I don’t know, yeah, more sessions would be good.” (Participant #12, 35yrs, quit smoking)  “…for me it was just, it just seemed a little short, there just didn’t seem to be an awful lot on there,...it was only, you see it didn’t seem like very much [encouragement] actually, it was only daily for a while and then it went to three, three times a week or something. Um, it didn’t seem like a lot, there didn’t seem like enough...” (Participant #1, 35yrs, quit smoking) |
| **5.2. Stop smoking medication** | **5.2.1. Attitudes and preferences** | | | “[when I relapsed] then there was a question ‘are you willing to do it with medicines?’ because I didn’t choose any medicines and I said ‘no’ and then it said ‘okay, we will find you another questionnaire’” (Participant #2, 26yrs, relapse and trying to quit)  “...I was using it [lozenges] initially [..] I stopped taking them and then it kept telling me I was gonna fail if I didn’t take them […] It didn’t sort of understand my change of circumstances that I’d changed my mind and didn’t want to use it anymore.. “Cos it seems that nicotine has got a bad, bad impact on the baby and... I’d lost my previous baby at 23 weeks so... ..so the sooner I could get out, get that out of my system the better really” (Participant #1, 35yrs, quit smoking).” (Participant #1, 35yrs, quit smoking)  “I had a concern about the website, [...] because it was asking over and over, are you using medicines and just giving tips about using medicines [...] I thought it was like a commercial thing [...] wants to promote the medicines” (Participant #2, 26yrs, relapse and trying to quit) |
|  | **5.2.2. Support for medication use** | | | “It asked the reason why so I put down why, but then there wasn’t any advice after that that I remember that was tailored to that particular type of NRT, for example I chose the lozenges, it wasn’t like it then in that session or future sessions came up with a suggestion list ....you’d have to know exactly what nicotine replacement therapy the woman was using and then like write new bits to suit all the different, know what I mean, another way in which the website’s could be a bit extended like suggestion list maybe of things like keep them in your handbag or your purse or your pocket when you’re leaving the house, or keep them here you know, little tips and things like that (Participant #12, 35yrs, quit smoking) |
| **Theme 6: Personal Contact** | | | | |
| **6.1. Peer-to-peer communication** | **6.1.1. Source of support and advice** | | | “… where someone, um, posts a question and then just the public answer it and there’s loads of, you know, confusing contradictory information whereas I know not to bother with those and to just look at, you know, NHS website or, you know, anything more official.” (Participant #7, 33, relapsed and trying to quit)  “...they’re all in the same boat and they might have ideas or advice that they can share, stuff that worked for them. [...] if I was on there and someone said they were struggling to give up their cigarette in the car, I could say “well what I did was”, you know, “and that’s how I gave up that one”, just sort of, it’s nice to share things like that isn’t it?” (Participant #6, 27yrs, quit smoking) |
|  | **6.1.2. Social support in difficult moments** | | | “..whereby people who stopped at the same time could actually encourage one another I think would be really helpful.”(Participant #1, 35yrs, quit smoking).  “I think, more support, like chat or email someone that you can sort of just say, “I’m not doing too good”, or even just to give positive feedback, just to say, “oh, I’m doing really well, thank you", sort of thing.” (Participant #11, 40yrs, trying to quit)  “I found it encouraging that other people were going through it […]…” (Participant #3, 20yrs, quit smoking) |
|  | **6.1.3. Understanding environment** | | | “Probably if I could give my biggest feedback would be whether to make it more of a community type website so that there’s ways of communicating more easily with other people..I assumed it would be when I joined, is that they would literally put you in, in touch with lots of other people who were giving up at the same time, and […] who were also pregnant who you could perhaps get to know one another and, I just thought that would be really beneficial.” (Participant #1, 35yrs, quit smoking)  “I just thought to keep it all [information and chat] in the one place, on the same website, it would be good, and you could speak to the same people that are using the same website […] somebody that’s had the same experiences, that understands, like there’s no point in speaking to somebody that hasn’t smoked. Yeah [e.g. someone who smoked], and has had the same experiences and understands that it is easy just to go and have one but then get back on track.” (Participant #11, 40yrs, trying to quit)  “I think I’d be more, I think I’d be more likely to speak to somebody in my position than the health advisor. ... I think if I was able to just join a community of some description I probably would actively join that. And talk to people about it, ‘cos it was also this obviously about the other side of the pregnancy as well...”. (Participant #1, 35yrs, quit smoking) |
|  | **6.1.4. Only for pregnant women wanting to quit** | | | “Um, yeah [I used forums] but not for the smoking side of it... No, not for giving up smoking, you couldn’t on places like that because it’s all judgment. […] because it’s all different people isn’t it? […] even if someone started a forum supporting each other to give up smoking it would soon be overtaken by people going “well you shouldn’t smoke anyway and don’t you know what...”, you know, kind of negative bits rather than supportive bits.” (Participant #6, 27yrs, quit smoking) |
| **6.2. Personal support** | **6.2.1. increase ISCI attractiveness** | | | “...sometimes it’s nice just to have that personal touch in it [..] that kind of, there’s that little personal Interaction…” (Participant #10, 24yrs, still smoking)  „...maybe even get [the people] that are behind the website you know, get them to do things like live chats […] people have got different attention spans so you know, they might sit and do a session but it might actually have more impact if they feel that they’ve got the attention of somebody that you know, has been part of this website and they can ask them questions..” (Participant #12, 35yrs, quit smoking) |
|  | **6.2.2. necessary support** | | | “I didn’t think it was going to help somebody telling me that they’re struggling and I were as well so I just wanted facts really.” (Participant #3, 20yrs, quit smoking)  “I thought I don’t know if it’s enough support for me, [...], I need more than online support, I think I need more like people, somebody to contact if I was like, there’s nobody there so if you’re doing it yourself it’s quite hard this isn’t going to be (Participant #11, 40yrs, trying to quit)  “... if somebody needed maybe a bit of extra support maybe there could be something that you could get telephone support if need be [...] maybe if somebody was really struggling and that was evident from every time they logged on they said that they’d had a cigarette and they were finding it really difficult, maybe there could be an alert system that somebody could then highlight these people that are on the programme who are using the website and then to get them further help and give that information to them as to where they can get that help from” (Participant #5, 25yrs, quit smoking) |
